# *Rhus chinensis* Mill. Fruits Ameliorate Hepatic Glycolipid Metabolism Disorder in Rats Induced by High Fat/High Sugar Diet

**DOI:** 10.3390/nu13124480

**Published:** 2021-12-15

**Authors:** Zihuan Wu, Qingqing Ma, Shengbao Cai, Yilin Sun, Yuanyue Zhang, Junjie Yi

**Affiliations:** 1 Faculty of Food Science and Engineering, Kunming University of Science and Technology, Kunming 650500, China; kmustwzh@163.com (Z.W.); caikmust2013@kmust.edu.cn (S.C.); syl115630519@163.com (Y.S.); 15911630471@163.com (Y.Z.); 2 School of Food Science and Technology, Jiangnan University, Wuxi 214122, China; maq_qing2018@163.com; 3 State Key Laboratory of Food Science and Technology, Jiangnan University, Wuxi 214122, China

**Keywords:** chronic disease prevention, energy metabolism, glycolipid metabolism, insulin regulation, obesity, *Rhus chinensis* Mill.

## Abstract

Hepatic glycolipid metabolism disorder is considered as one of the key factors in the pathogenesis of many chronic diseases. The objective of this study was to investigate the protective effect and underlying mechanisms of *Rhus chinensis* Mill. fruits against hepatic glycolipid metabolic disorders in rats induced by a high fat/high sugar diet. Results showed that ethanol extract, especially at a dose of 600 mg/kg b.w., could effectively ameliorate glycolipid metabolic disorders in rats. The biochemical indexes, including CAT, GSH and HOMA-IR, were significantly improved by the administration of ethanol extract. Immunohistochemistry and Western blot analysis revealed that ethanol extract up-regulated the expression levels of PI3K/AKT, PPAR-α, and the phosphorylation of IRS1 and AMPK proteins, and down-regulated the expressions of SREBP-1 and FAS proteins in the liver, which are closely related to hepatic glycolipid metabolism. Those findings suggested that *R. chinensis* Mill. fruits could be developed as functional foods and/or nutraceuticals for preventing or controlling some chronic diseases related to hepatic glycolipid metabolism disorder.

## 1. Introduction

Glycolipid metabolism, as an important biological energy supply of the human body, plays an important role in life activities. However, the disorder of glycolipid metabolism will lead to a series of chronic metabolic diseases, such as diabetes, fatty liver, hyperlipidemia, obesity, arteriosclerotic, and cardio-cerebrovascular disease [1]. The International Federation Atlas (2018) reported that more than 415 million people have diabetes, and the number is expected to rise to 642 million by 2040 [2]. Meanwhile, epidemiological studies showed that the global prevalence of nonalcoholic fatty liver disease (NAFLD) is about 25% [3]. Those chronic metabolic diseases impose not only a heavy burden on health care systems around world, but also remarkably affect the life quality of patients [4,5]. Therefore, effectively preventing and/or ameliorating glycolipid metabolism disorder has become an important research direction [6].

Previous studies have indicated that the liver is the important location of glycolipid metabolism conversion, which is closely related to the normal regulation of blood glucose and lipids and the formation of a variety of chronic metabolic diseases [7,8]. Therefore, hepatic glycolipid metabolism is considered to play a decisive role in the prevalence of metabolic diseases, such as diabetes, obesity, and fatty liver disease [8,9]. After consuming a large amount of sugar and fat, the concentration of blood glucose increases, which stimulates the pancreas islet to secrete insulin to increase glucose uptake by the liver [10,11]. Excessive insulin will guide the liver to convert excess glucose into lipids, and, at the same time, the body also intakes large amounts of lipids from the diet, which eventually exceed the liver metabolic capacity and induce abnormal accumulation of lipids in the liver [12,13]. Lipid accumulation in hepatocytes is the hallmark of NAFLD and an important factor that can induce lipid peroxidation, insulin resistance (IR), changes in energy metabolism, hepatocyte damage and inflammation, thus leading to obesity, diabetes, fatty liver, and other diseases [13,14].

Insulin controls blood glucose by regulating insulin receptor substrate 1 (IRS1), phosphoinositide 3 kinase (PI3K) and protein kinase B (AKT); the activation of AMP-activated protein kinase (p-AMPK) and peroxidase proliferative receptor-α (PPAR-α) promotes fatty acid β-oxidation to regulate lipid metabolism [2,15]. Therefore, a sample that can regulate those key target proteins may effectively improve glycolipid metabolism disorder in the human body. Thus, exploiting natural products or functional foods that can prevent and/or improve glycolipid metabolism disorders with few side effects is considered as one of the necessary and important strategies to deal with the issue of glycolipid metabolism disorder [9]. Many natural products have been proven to exhibit a good improving effect against glycolipid metabolism disorder, such as kiwi fruit seed oil, yam polysaccharide, betaine, and phenolic compounds [6,16].

*Rhus chinensis* Mill. belongs to the *Anacardiaceae* family and is an important economic tree in Asia that can be used as a raw material for pharmaceutical and industrial dyes [17]. The leaves, roots and fruits of this plant can be used as traditional Chinese medicines to treat diarrhea, malaria, hepatitis, and jaundice [18]. Moreover, the fruit oil of this plant was approved by the Chinese authorities as a new food ingredient for daily consumption in 2013. Previous studies have shown that *R. chinensis* Mill. fruits contain large amounts of phenolic compounds, and the phenolic extract exhibited good inhibitory effect against biological oxidation [4]. However, to the best of our knowledge, few studies have been performed in vivo to determine the effect of *R. chinensis* Mill. fruits on hepatic glycolipid metabolism disorder. Therefore, this study aimed to investigate the effect of *R. chinensis* Mill. fruits against hepatic glycolipid metabolism disorder in rats induced by a high-fat and high-sugar diet (HFHSD). Moreover, the underlying mechanisms were illuminated by the immunohistochemistry and Western blot analyses of several critical proteins involved in regulating the glycolipid metabolism in the liver.

## 2. Materials and Methods

### 2.1. Chemical, Reagents and Sample

All kits used in this work were obtained from Nanjing Jiancheng Bioengineering Institute (Nanjing, China), except insulin (ELISA kit, Thermo) and blood glucose (GA-3, Sinocare). All antibodies were procured from Abcam (Cambridge, UK). All other reagents used were of analytical grade.

### 2.2. Extract Preparation and Analysis

*R. chinensis* Mill. fruits were purchased from Kunming Plant Classification Biotechnology Co., Ltd. and stored at −30 °C. Fruit powder was ultrasonically extracted by 80% ethanol with the ratio of material to liquid at 1:5 (*m*/*v*). Phytochemical composition of the ethanol extract was analyzed by using UHPLC-ESI-HRMS/MS based on the previous method [3,4]. Results showed that the extract was rich in phenolic compounds, and gallic acid, quercetin-3-*O*-rhamnoside, and myricetin-3-*O*-rhamnoside were the main detected phenolic substances.

### 2.3. Animals and Treatment

Twenty-four Sprague-Dawley (SD) male rats (specific pathogen-free, 6–8 weeks old), with body weights of approximately 200 g ± 5%, were used in this study. The experimental animals were purchased from Liaoning Changsheng Biotechnology Co., Ltd., Liaoning, China (Certificate No.: SCKK 2015–0002). All rats were adapted to the new environment for one week before starting the experiment. Then, the rats were divided into four groups (*n* = 6) as follows: groups C (control group, normal diet), M (model group, HFHSD), AL (low dose group, 200 mg/kg of ethanol extract + HFHSD), and AH (high dose group, 600 mg/kg of ethanol extract + HFHSD). The doses of ethanol extract applied in the current work were according to the results of the previous toxicity study [17] and our preliminary experiment. Basic feed was purchased from Kunming Medical University, and high-fat and high-sugar feed (50% basic feed, 15% lard oil, 10% yolk powder, 10% fructose, 12% corn oil, 2% fish meal, and 1% cholesterol) was purchased from Kunming Zanna Biotechnology Co., Ltd. (Kunming, Yunnan, China). The experiment lasted for 12 weeks, and the weights of rats were measured weekly. The conditions for rat feeding were referred from a previous paper [17]. All animal procedures were carried out in strict accordance with the National Institute of Health Guide for the Care and Use of Laboratory Animals, and approved by the Ethical Committee for Animal Experimentation of Kunming University of Science and Technology.

### 2.4. Biochemical Parameters

At the end of the experiment, the rats were weighed after fasting for 8 h. A vacuum blood vessel with lithium heparin was used to collect blood from the celiac artery of each rat. Then, all blood samples were centrifuged at 2000× *g* and 4 °C for 5 min. Plasma was prepared from blood by centrifuging at 1800× *g* (4 °C, 5 min) and used for the determination of triglyceride (TG), total cholesterol (TC), low-density lipoprotein cholesterol (LDL-C), high-density lipoprotein cholesterol (HDL-C), insulin and blood glucose with the corresponding kit according to the manufacturer’s instruction. Subsequently, the tissue homogenate of the liver was prepared as follows: an amount of 0.5 g of the liver tissue was cut off, placed in 4.5 mL of normal saline, and homogenized by using an ultrasonic cell crusher. After complete homogenization, all homogenate samples were centrifuged at 10,000× *g* and 4 °C for 5 min. The supernatant was used to determine the TG, TC, catalase (CAT), superoxide dismutase (SOD), reduced glutathione (GSH) and malondialdehyde (MDA) by using the corresponding commercial kit. The homeostasis model assessment of insulin resistance (HOMA-IR) was used to determine IR according to the following formula: fasting plasma glucose (mmol/L) × fasting plasma insulin (mIU/L)]/22.5.

### 2.5. Magnetic Resonance Imaging

The day before the end of the experiment, the rats were anesthetized and scanned via magnetic resonance imaging (MRI) [19,20]. All data were obtained by using a 3.0-Tesla system (HDXT 3.0T, General Electric Company, Wisconsin, USA) with orthogonal matrix and head array coils for signal reception. The abdominal and subcutaneous fat distribution was scanned by using routine Cor-3D-Fiesta-C, and the parameters were as follows: TE: 4.8 ms, Flip angle: 17.0 cm, Slice thickness: 1.8 mm, Freq: 512, Phase: 512, Next: 3, Phase FOV: 1. In-phase and out-of-phase (IP-OP) MRI with 3D gradient echo sequence was also used to scan the abdominal fat distribution [21]. The detailed parameters of IP-OP were as follows: T1-in phase: TR: 270 ms, TE: 2.5 ms, FOV: 13.0 cm, Slice thickness: 1.0 mm, Freq: 192, Phase: 128, Next: 14, Phase FOV: 0.6; T1-out of phase: TR: 245 ms, TE: 3.2 ms, FOV: 13.0 cm, Slice thickness: 1.0 mm, Freq: 192, Phase: 128, Next: 14, Phase FOV: 0.6.

### 2.6. Histopathological and Immunohistochemical Analyses

The liver lobule, adipose tissue, and pancreatic tissue were immersed in a 4% paraformaldehyde solution and then embedded in paraffin. The histopathological examination of adipose tissue was performed by using hematoxylin–eosin (H&E) staining. The liver glycogen was stained with the periodic acid–Schiff technique. The section results were observed by using a microscope (Olympus IX83, Tokyo, Japan). Immunohistochemistry analysis was performed as follows: the section was placed in dimethyl benzene to deparaffinate, rehydrated with graded alcohol, and blocked with 5% bovine serum albumin (BSA) for 10–30 min [22]. Phosphate buffer saline (PBS) was used to wash the tissue sections after incubation with primary antibodies and then incubated with a secondary antibody. Then, all sections were washed and observed under the Olympus IX83 microscope.

### 2.7. Western Blot Analysis

The middle lobe of the liver was placed in lysis buffer (containing protease and phosphatase inhibitor) and homogenized by using an ultrasonic cell crusher (scientz-II D, Ningbo Scientz Biotech Co., Ltd., Ningbo, China). Then, the targeted proteins were separated by sodium dodecyl sulfate-polyacrylamide gel electrophoresis, and then blotted onto a nitrocellulose (NC) membrane. Thereafter, the NC membrane was incubated with the corresponding primary and secondary antibodies. After incubation, proteins were examined by using a VILBER Fusion FX7 imaging system (Vilber Lourmat, Marne-la-Vallée, France) with an enhanced chemiluminescent detection reagent (Millipore, Billerica, MA, USA).

### 2.8. Statistical Analysis and Evaluation of the Protein Expression via Image Pro Plus

The results were expressed as mean ± standard error (S.E.) and analyzed by using one-way ANOVA in the Origin 9.0 software (OriginLab; Northampton, MA, USA). Tukey’s test was applied to determine the significance of differences (*p* < 0.05), while in the Western blot analysis’s experiment, we used the Kruskal–Wallis test and calibrated the *p* value with false discovery rate in the GraphPad Prism 8 (GraphPad Software Inc., La Jolla, CA, USA). All slices were observed in five places, that is, four visual fields were randomly selected around the slices, plus the visual field in the middle position. The average calculated by these five fields of vision is the value of this slice. The immunohistochemistry was evaluated by using the Image-Pro plus 6.0 (IPP) software (Media Cybernetics Inc.; Rockville, MD, USA) as follows: optical density was first calibrated, and the area of interest was set (hue, 0–0; saturation, 0–255; intensity, 0–255). The density sum and area sum parameters were measured to calculate the mean density (density mean = density sum/area sum, and the number of adipocytes were calculated manually) [23,24].

## 3. Results and Discussion

### 3.1. Analysis of Biochemical Indicators

**Figure 1:**
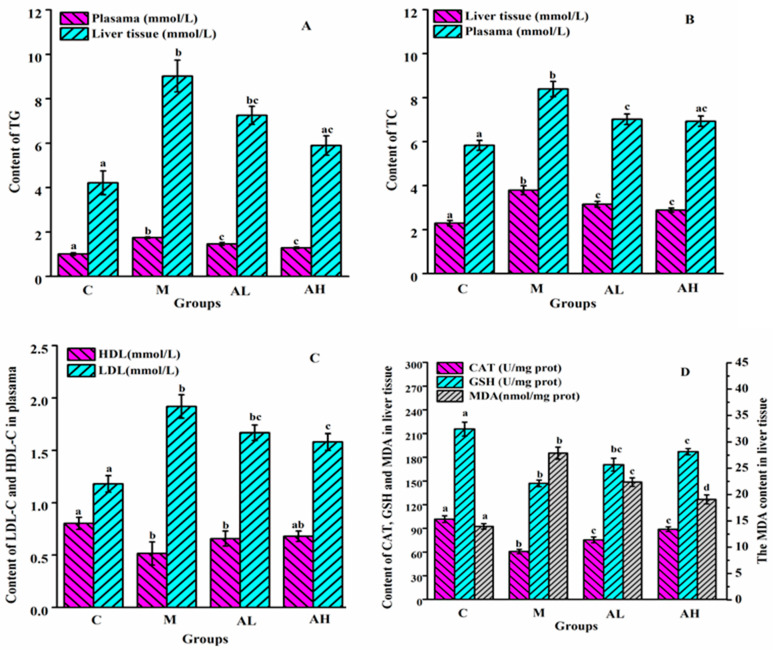
Effects of ethanol extract from *R. chinensis* Mill. fruits on the biochemical parameters of plasma and liver tissue induced by a high fat/high sugar diet. (**A**) Triglyceride (TG)contents in plasma and liver tissue. (**B**) Total cholesterol (TC) contents in plasma and liver tissue. (**C**) Low-density lipoprotein cholesterol (LDL-C) and High-density lipoprotein cholesterol (HDL-C) contents in plasma. (**D**) Catalase (CAT), Reduced glutathione (GSH) and Malondialdehyde (MDA) contents in liver tissue. C: Control group, M: Model group, AL: Low dose ethanol extract group (200 mg/kg), AH: High dose ethanol extract group (600 mg/kg). Data were expressed as the mean ± SE (*n* = 6). Different letters indicate significant differences (*p* < 0.05).

Dysregulation of glucose and lipid metabolism in the liver is a potential cause of chronic metabolic diseases, accompanied by dyslipidemia, declined antioxidant defense systems, and enhanced lipid peroxidation [25]. In the present work, the model rats displayed obvious abnormality of lipid metabolism, and impaired endogenous antioxidant system and IR according to the results of biochemical indexes in plasma and liver tissue (Figure 1). In plasma, the contents of TG, TC and LDL-C in group M were significantly higher than those in group C (*p* < 0.05), while the content of HDL-C in group M was remarkably lower than that in group C (*p* < 0.05). Moreover, in liver, the contents of TG, TC and MDA in group M were also elevated significantly when compared with those in group C (*p* < 0.05), while the contents of CAT and GSH in group M were remarkably lower than those in group C (*p* < 0.05). Those results observed in the present work were in consistent with the previous findings that obvious lipid abnormalities were found in the plasma and liver of obese rats or mice induced by a high-fat diet [26,27].

Compared with the group M, the plasma and hepatic lipid parameters in both AL and AH groups were significantly improved, especially those in group AH (*p* < 0.05). Numerous previous studies also reported that the lipid profiles of plasma and liver in obese rats or mice induced by a high-fat diet improved obviously after treating with various plant extracts [26,28]. It is found that HFHSD changes the metabolism of lipid-lipoprotein, resulting in increased plasma TC and LDL levels, as well as decreased HDL levels, which further drives the development of atherosclerosis and increases the risk of cardiovascular diseases [29]. In the human body, cholesterol is primarily circulated by HDL-C and LDL-C. LDL-C carries cholesterol from liver to tissues. LDL-C may adhere to the arterial wall and increase the morbidity of atherosclerosis when it is oxidized into oxidized low-density lipoprotein (ox-LDL) during transportation [30]. However, HDL-C can not only transport excess cholesterol from tissues to the liver, but also infiltrate into the artery wall to clear adhered cholesterol from arteries to reduce the risk of cardiovascular diseases [31]. In the present work, the administration of ethanol extract, especially at high dose, could significantly counteract the changes of LDL-C and HDL-C in obese rats, indicating that the ethanol extract of *R. chinensis* Mill. fruits may be potentially beneficial to preventing cardiovascular diseases, which is needed to be proved in the future experiment.

The liver is a key organ in human lipogenesis, gluconeogenesis, and cholesterol metabolism. Previous studies have confirmed that a high level of hepatic oxidative stress occurred in model mice or rats induced by high fat/high sugar diets, which can deplete the endogenous antioxidant system, and thereby result in many chronic diseases such as complications of obesity, diabetes, and NAFLD [8,9]. The main endogenous antioxidant system in the liver consists of CAT, GSH, and so on, which can maintain the normal oxidation level of the body to protect cells against oxidative stress-related damage. Meanwhile, the decrease of endogenous antioxidant enzymes or substances will lead to the increase of lipid peroxidation. The main product of lipid oxidation is MDA, which damages cell membrane and induces cross-linking polymerization of nucleic acids and proteins, thus leading to their inactivation. Figure 1D presents that the levels of CAT and GSH in group M were significantly lower than those in group C (*p* < 0.05), while the content of MDA was remarkably higher than that in group C (*p* < 0.05). Those results suggest that the endogenous antioxidant system was compromised by HFHSD. However, the antioxidant capacities of the ethanol extract-treated groups were improved remarkably, especially that in group AH (*p* < 0.05), thereby indicating that ethanol extracts from *R. chinensis* Mill. fruits could restore the endogenous antioxidant capacity of the liver to reduce the risks of diseases related to oxidative stress and lipid peroxidation caused by HFHSD [32,33]. Previous studies also demonstrated that improving GSH and CAT activity could alleviate the negative impact of glycolipid metabolism disorder [34].

### 3.2. Fat Distribution and Insulin Resistance

**Figure 2:**
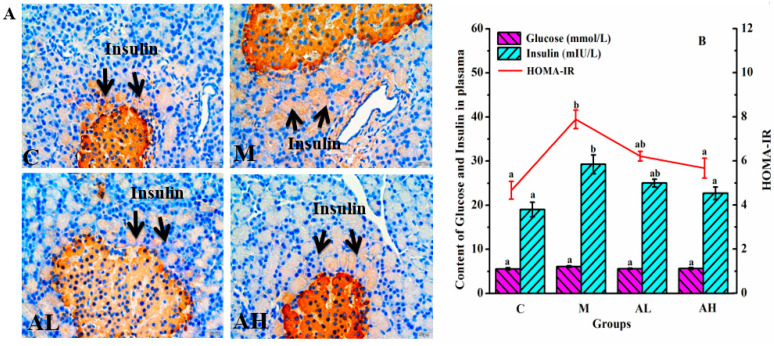
Immunohistochemical staining of insulin in pancreas islets and homeostasis model assessment of insulin resistance (HOMA-IR) in rats. (**A**) Immunohistochemical expression of insulin in rats. (**B**) HOMA-IR. The yellow part in Figure 2A represents insulin. C: Control group, M: Model group, AL: Low dose ethanol extract group (200 mg/kg), AH: High dose ethanol extract group (600 mg/kg). Data were expressed as the mean ± SE (*n* = 6). Different letters indicate significant differences (*p* < 0.05).

**Figure 3:**
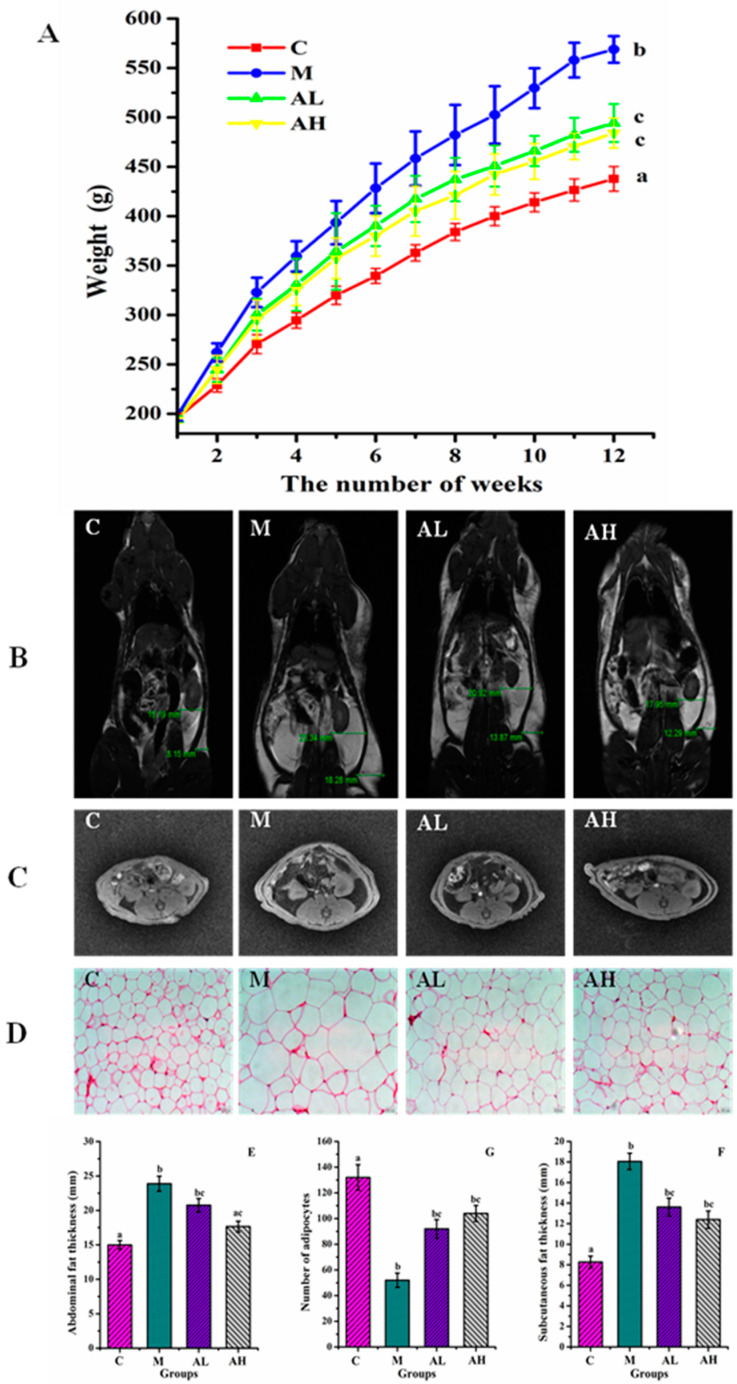
Body weight changes, distribution, and thickness of abdominal and subcutaneous fat in rats detected by magnetic resonance imaging (MRI), and the adipocyte number changes in rats. (**A**) Body weight changes of rats over 12 weeks (*n* = 6). (**B**) Distribution of abdominal and subcutaneous fat. (**C**) Results of in-phase and out-of-phase magnetic resonance imaging. (**D**) H&E staining of adipose tissue (*n* = 6). (**E**) Thickness of abdominal fats (*n* = 3). (**F**) Thickness of subcutaneous fats (*n* = 3). (**G**) Adipocyte number changes (*n* = 6), which were calculated manually. Data in (**B**,**C**) were all expressed as mean ± SE. The white component in Figure 3B and the black component in Figure 3C is adipose tissue. C: Control group, M: Model group, AL: Low dose ethanol extract group (200 mg/kg), AH: High dose ethanol extract group (600 mg/kg). Data were expressed as the mean ± SE. Different letters indicate significant differences (*p* < 0.05).

Diets rich in glucose and/or lipids increase blood glucose and blood free fatty acid (FFA) levels, which stimulates insulin secretion by islet cells and then causes hyperinsulinemia, and thereby results in reduced insulin-mediated glucose uptake and increased lipid synthesis [13,35]. Furthermore, HFHSD and high insulin can lead to the storage of large amounts of energy in adipocytes by accumulating lipids in the form of triglyceride, thus resulting in the imbalance of glycolipid metabolism [35,36]. Insulin signaling is chronically disrupted in adipose tissue of model rats, and the blood glucose and FFA levels may increase when fat decomposition is performed [36,37], thus aggravating the disorder of glycolipid metabolism [38,39]. The main feature of IR is attributed to the insensitivity of cells or tissues toward insulin, which is the main pathological factor leading to obesity, diabetes and NAFLD, and the HOMA-IR is often used as the evaluation index [2,40]. The immunohistochemical of the islet tissue and biochemical criterion results are shown in Figure 2A. The results showed that HFHSD significantly induced the increase in insulin secretion of the islet in group M (*p* < 0.05), while no significant change in plasma glucose level was observed (*p* < 0.05). However, compared with group C, the IR index (HOMA-IR) in group M increased significantly (*p* < 0.05). Figure 3 presents that the average body weight of rats in group M is significantly higher than those of the other three groups (*p* < 0.05), especially at the late stage of the experiment. The average body weight of rats in group AL and AH were significantly suppressed when compared with that of group M (*p* < 0.05).

Therefore, by combining the results of Figure 2, we can conclude that ethanol extract of *R. chinensis* Mill. fruits can effectively improve the IR in rats induced by HFHSD, especially in the group AH. Considerable studies have demonstrated that fat distribution is more important than fat itself [41]. Excessive abdominal fat is more harmful to health than subcutaneous fat, and is associated with the incidence of diseases, including cardiovascular disease and type 2 diabetes [42]. The accumulation of adipose tissue in the abdominal cavity might squeeze the internal organs, compress the celiac artery, and compromise venous flow rate, and thereby result in stenosis and occlusion of the vessel, or other dysfunctions of visceral organs [43]. The fat distributions in rats in this work were evaluated by using MRI as presented in Figure 3B,C. Both abdominal and subcutaneous fats of rats in group M increased significantly when compared with those in group C (*p* < 0.05) (Figure 3E,G). For group AH, the thickness of abdominal fats of rats was similar to that of rats in group C, and was significantly reduced when compared with those in group M (*p* < 0.05) (Figure 3E,G). However, the thickness of both abdominal and subcutaneous fat in group AL was not significantly lower than that of rats in group M (*p* > 0.05) (Figure 3B,E,F). Figure 3C clearly indicates that the volume of the combined abdominal fat in group AL is smaller than that in group M, and the seemingly paradoxical results of fat thickness in group AL may be due to the body position of rats. Moreover, the subcutaneous fat thickness of rats in both AL and AH groups showed a downward trend compared with the M group (Figure 3G). Increased adipocyte size is usually an important cause and characteristic of IR [13,44]. Figure 3D illustrates that the adipocyte size in group M is obviously larger than that in group C, and the number of adipocytes in group M is significantly lower than that in group C at the same magnification (Figure 3F). Compared with group M, the adipocyte size in both AL and AH groups was significantly decreased (*p* < 0.05). Therefore, by combining the results of Figure 3, we can conclude that ethanol extract of *R. chinensis* Mill. fruits can effectively inhibit the increase of abdominal, subcutaneous fats and IR in rats induced by HFHSD, and thereby significantly ameliorate the disorder of glycolipid metabolism.

### 3.3. Immunohistochemistry and Protein Expression Analysis

**Figure 4:**
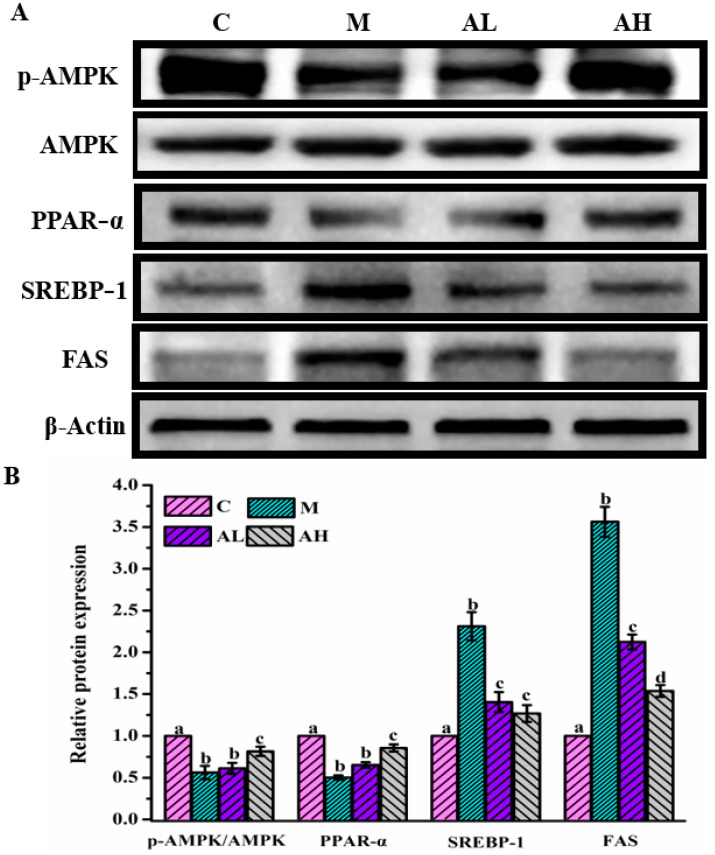
The effect of ethanol extract from *R. chinensis* Mill. fruits on the expression of several key proteins related to lipid metabolism in the liver, including amp-activated protein kinase (AMPK), phosphorylated amp-activated protein kinase (p-AMPK), peroxidase proliferative receptor-α (PPAR-α), sterol regulatory element-binding protein1 (SREBP-1), and fatty acid synthase (FAS). (**A**,**B**) represent the lipid pathways and the relative quantity of protein, respectively. The relative expressions of above proteins were quantified by normalization with group C and compared with β-actin. C: Control group, M: Model group, AL: Low dose ethanol extract group (200 mg/kg), AH: High dose ethanol extract group (600 mg/kg). Data were expressed as the mean ± SE (*n* = 6). Different letters indicate significant differences (*p* < 0.05).

A long-term HFHSD can cause hepatic glycolipid metabolism disorder, while inhibiting the disorder occurrence can effectively prevent the occurrence of chronic metabolic disease [45]. Based on the results of glycolipid metabolism observed in plasma and liver (Figure 1 and Figure 2), HFHSD-induced model rats exhibited obvious abnormality of glycolipid metabolism, and ethanol extract treatment could significantly alleviate this abnormality, which suggested that the ethanol extract may regulate the expressions of some proteins related to glycolipid metabolism, since the liver is one of the main organs responsible for glycolipid metabolism, including absorption, transportation, catabolism and anabolism of lipids and carbohydrates [8]. Lipid metabolism in the liver is mainly regulated by a series of proteins, such as AMPK, PPAR-α, sterol regulatory element-binding protein1 (SREBP-1), and FAS [8,15]. AMPK is an important protein that regulates energy metabolism. Activating AMPK (p-AMPK) can reduce triglycerides and cholesterol synthesis, and increase fatty acid oxidation in the liver, which is eventually beneficial for ameliorating the disorder of glycolipid metabolism [15,46]. Figure 4 illustrates that the expression of p-AMPK is significantly reduced in group M compared with that in group C (*p* < 0.05), whereas its expression levels in all ethanol extract-treated groups were remarkably up-regulated, especially in group AH (*p* < 0.05), which was similar to that in group C (*p* > 0.05). SREBP-1, a downstream protein of AMPK, can bind to a specific protein molecule in the nucleus that upregulates the expression of FAS protein to increase the synthesis of fatty acids [47]. It is found that p-AMPK can inhibit the cleavage and nuclear translocation of SREBP-1, leading to reduced lipogenesis and lipid accumulation [48]. The FAS protein is a key enzyme for de novo synthesis of free fatty acid which is a raw material for fat synthesis [49]. Therefore, the down-regulation of FAS expression was considered as an effective target for preventing and/or controlling glycolipid metabolism disorder. As shown in Figure 4, the expression of SREBP-1 in group M was significantly higher than those in the other three groups (*p* < 0.05). Meanwhile, the results shown in Figure 4 indicated that the expression of FAS in group M was significantly higher than that in group C (*p* < 0.05), whereas a significant down-regulation of FAS expression was observed in all ethanol extract-treated groups compared with that in group M (*p* < 0.05). This result showed that ethanol extract of *R. chinensis* Mill. Fruits can effectively reduce the synthesis of fatty acids. Bae et al. (2019) also reported that the combination of diallyl disulfide and green tea exhibited a good anti-obesity by down-regulating the expressions of SREBP-1 and FAS [50]. Another study found that alisol A could upregulate p-AMPK and suppress SREBP-1 in obese C57BL/6 mice induced by a high-fat diet [48].

Moreover, previous studies have also demonstrated that PPAR-α involved in energy expenditure could enhance the oxidation of fatty acids by activating the liver-specific carnitine transporter, which is responsible for transporting fatty acids from the cytosol into the mitochondria for subsequent β-oxidation, thus reducing the accumulation of lipid in tissues [51,52]. Figure 4 shows that the expression of PPAR-α in group M decreased significantly when compared with that in group C (*p* < 0.05), whereas that in high-dose ethanol extract-treated groups improved remarkably (*p* < 0.05). This result was consistent with the findings reported by many previous studies with many different food materials [53,54]. Nanako Nihei et al. found that dietary α-cyclodextrin could increase PPAR-α to improve lipid metabolism in mice fed with a high-fat diet [53]. An et al. (2018) also reported that a phytol-enriched diet ameliorated obesity and its related metabolic abnormalities by activating PPAR-α in the liver and brown adipose tissue of male C57BL/6 mice [55]. Moreover, a clinical trial also found that PPAR-α expression was significantly up-regulated by oleoylethanolamide to reduce the body weight of obese people [54].

**Figure 5:**
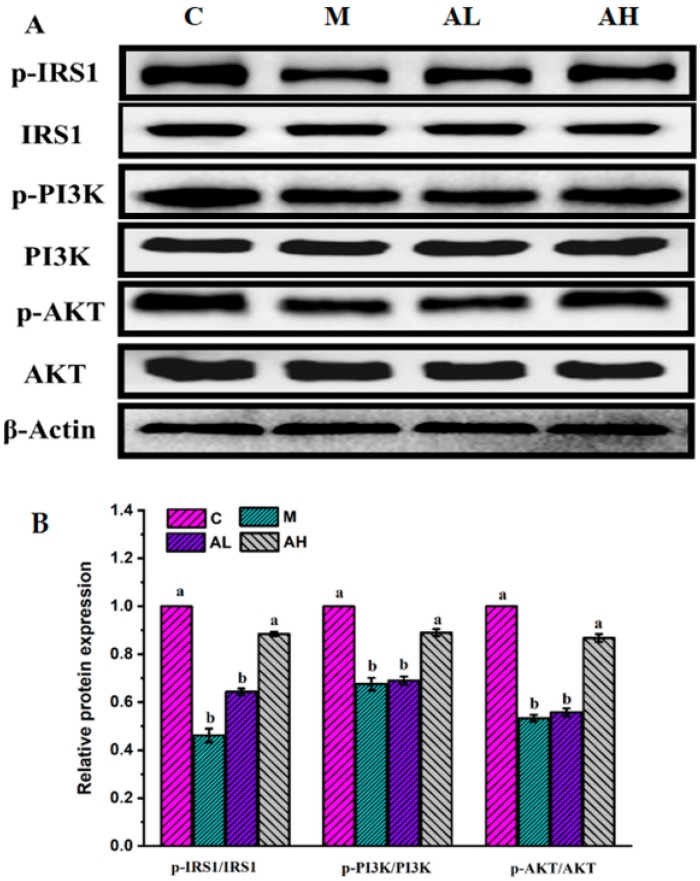
The effect of ethanol extract from *R. chinensis* Mill. fruits on the expression of several key proteins related to glycometabolism in the liver, including insulin receptor substrate-1(IRS1), phosphorylated insulin receptor substrate-1 (p-IRS1), phosphatidylinositol 3 kinase (PI3K), phosphorylated phosphatidylinositol 3 kinase(p-PI3K), protein kinase B(AKT), and phosphorylated protein kinase B (p-AKT). (**A**,**B**) represent the glucose pathways and the relative quantity of protein, respectively. The relative expressions of above proteins were quantified by normalization with group C and compared with β-actin. C: Control group, M: Model group, AL: Low dose ethanol extract group (200 mg/kg), AH: High dose ethanol extract group (600 mg/kg). Data were expressed as the mean ± SE (*n* = 6). Different letters indicate significant differences (*p* < 0.05).

**Figure 6:**
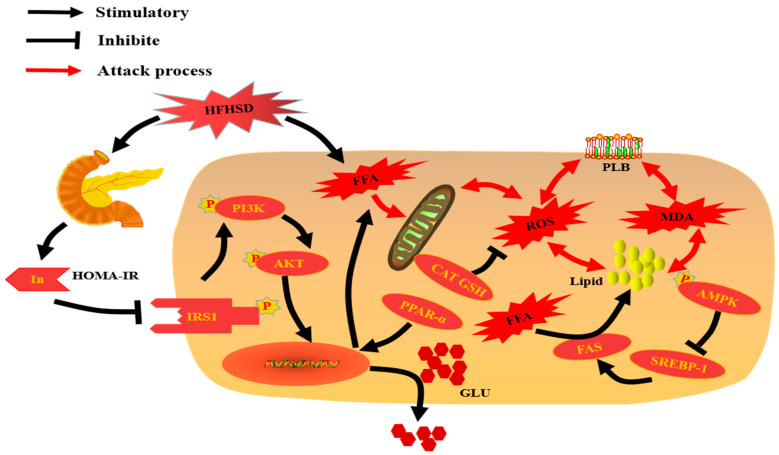
Schematic diagram of the main mechanism of glycolipid metabolism disorder pathways investigated in the present work. CAT: catalase; GSH: reduced glutathione; FFA: free fatty acid; ROS: reactive oxygen species; MDA: malonaldehyde; AMPK: amp-activated protein kinase; PPAR-α: peroxidase proliferative receptor-α; HOMA-IR: homeostasis model assessment of insulin resistance; IRS1: insulin receptor substrate 1; PI3K, phosphoinositide 3 kinase; AKT: protein kinase B; SREBP-1: sterol regulatory element-binding protein1; HFHSD: high-fat and high-sugar diet; GLU: glucose; PLB: phospholipid bilayer; FAS: fatty acid synthase.

Glycogen synthesis from glucose takes place in many tissues, but it is particularly important in the liver and is regulated by insulin [56]. Therefore, we further explored potential mechanism of glucose metabolism in the liver. It is widely acknowledged that impaired IRS1 phosphorylation will reduce IRS1-associated PI3K activities to affect glycogen synthesis and glucose uptake in the liver through the PI3K/AKT signaling pathway, and eventually induce hepatic IR [2,40]. Impaired glucose metabolism may result from impaired IRS1 and the PI3K/AKT signaling pathway in the liver [40]. Moreover, IRS1, the receiving insulin signal, is necessary for controlling downstream of the PI3K/Akt pathway [2,40]. If the signal of insulin cannot be effectively transmitted to IRS1 or activated, it cannot activate PI3K for subsequent glucose metabolism signal transduction [2,40]. AKT normally regulates glucose metabolism through downstream proteins and nuclear receptors, while resting PI3K cannot activate AKT and thereby results in a serious disturbance of glucose metabolism [2,40]. In this work, p-IRS1 was effectively activated in group C, whereas its expression in group M was significantly down-regulated (*p* < 0.05) (Figure 5). Meanwhile, downstream proteins PI3K and AKT were reduced in group M compared with those in group C (*p* < 0.05) (Figure 5). By comparing with group M, the expressions of p-IRS1 and downstream proteins in the PI3K/AKT signal pathway were remarkably improved in the high dose of ethanol extract-treated group (*p* < 0.05) (Figure 5). Yange Wei et al. found that dietary ginsenoside Rg5 could increase IRS-1/PI3K/AKT to activate glucose metabolism and attenuate hepatic IR in db/db mice [2]. Kaiping Wang et al. also reported that dendrobium officinale polysaccharide had a remarkable hypoglycemic effect in type 2 diabetes mellitus mice induced by streptozotocin, which might be associated with the activity of glucose metabolism enzymes via activating the IRS1/PI3K/Akt signaling pathway in mice [40]. All those results suggested that the rats fed with HFHSD were insensitive to insulin signaling, which in turn induced more insulin secretion and resulted in the over synthesis of fat [57]. The results suggested that ethanol extracts from *R. chinensis* Mill. fruits could improve insulin utilization by regulating the expressions of p-IRS1 and PI3K/AKT pathway proteins to improve the glucose metabolism. The main mechanisms of glycolipid metabolism disorder induced by HFHSD investigated in the present work are outlined in Figure 6.

Therefore, according to the results obtained in the present work (Figure 4), the ethanol extract from *R. chinensis* Mill. fruits may alleviate hepatic glycolipid metabolic disorders induced by HFHSD via regulation of the expressions of several key proteins involved in glycolipid metabolism (p-AMPK, PPAR-α, SREBP-1, FAS, p-IRS1 and PI3K/AKT) in the liver. Moreover, our results suggest that *R. chinensis* Mill. fruits may have the potential to prevent or control diseases related to glycolipid metabolism, such as obesity, diabetes, NAFLD, and atherosclerosis, which needs a comprehensive investigation in the future.

## 4. Conclusions

This study demonstrated that ethanol extract of *R. chinensis* Mill. fruits could effectively improve hepatic glycolipid metabolism disorders in rats induced by HFHSD, wherein the high dose of ethanol extract exhibited a better effect. The protective effect of ethanol extract from *R. chinensis* Mill. fruits against hepatic glycolipid metabolism disorders were regulated through the AMPK/SREBP-1/FAS, IRS1, and PI3K/AKT signaling pathways. Meanwhile, ethanol extract could restore innate antioxidant enzymes to protect the tissues and cells from the oxidative damage caused by hepatic glycolipid metabolism disorders. Those findings suggested that *R. chinensis* Mill. fruits could be developed as functional foods or nutraceuticals for preventing or controlling diseases related to the disorders of glycolipid metabolism.

## 5. Limitations of Study

While we demonstrate in this study that the high dose of ethanol extract of *R. chinensis* Mill. fruits could improve hepatic glycolipid metabolism disorders effectively in rats, there are several key limitations that should be noted. First, we only tested a mixture from *R. chinensis* Mill. fruits for the improvement of glycolipid metabolism disorders in our rat model. This experiment covers all ingredients, and it would be interesting to determine what monomer compound has this capability. Another limitation of this study is that it only analyses hepatic glycolipid metabolism, and it would be interesting to characterize the extract improving the glycolipid metabolism disorders activating capacity. Additionally, we only evaluated the extract repair glycolipid metabolism and the other efficacy of the extract via other animal models should be included in future studies.

## Data Availability

The data that support the findings of this study are available from the corresponding author upon reasonable request.
